# Single Oral Dose Toxicity Study of an Alcohol Extract of Bumblebee, *Bombus ignitus* Larvae in Rats

**DOI:** 10.5487/TR.2009.25.2.107

**Published:** 2009-06-01

**Authors:** Mi Young Ahn, Jea Woong Han, Hyung Joo Yoon, Hae Chul Park, Wan Tae Chung

**Affiliations:** 17 Department of Agricultural Biology, National Institute of Agricultural Science and Technology, RDA, 61 Seodun-Dong, Kwonsun-gu, Suwon, 441-100 Korea; 27 National Institute of Animal Science, RDA, Suwon, 441-350 Korea

**Keywords:** *B. ignitus* larvae extract, Single oral dose toxicity test

## Abstract

The alcohol extract of the larvae of *Bombus ignitus*, otherwise known as the Bumblebee, was orally administered to rats at doses of 0, 0.04, 0.2, 1 or 2 g/kg as a single oral dose. There were no observed clinical signs or deaths related to treatment in all the groups tested. Therefore, the approximate lethal dose of the alcohol extract of *B. ignitus* was considered to be higher than 2 g/kg in rats. Mild decreases in body weight gain in male rats were observed dose-dependently within the *B. ignitus* treated groups over 2 weeks. Throughout the administration periods, no significant changes in diet consumption, ophthalmologic findings, clinical pathology (hematology, clinical chemistry and coagulation) or gross pathology were detected. Minor changes in male rats were found with in the hematological parameters in groups treated with the 0.04 g/kg, 1 g/kg or 2 g/kg of *B. ignitus* larvae extract, however, all the changes observed were within the physiological range. From these results, it was concluded that there was no evidence of specific toxicity related to the ingestion of alcohol extract of *B. ignitus* larvae.

## INTRODUCTION

The medicinal and nutritional applications of honey and other hive products including bee larvae are well known (Meda *et al*., 2004). Bumblebees of the Apidae family are distributed worldwide including alpine regions (Galen and Cuba, 2001). Twenty one species belonging to seven subgenera in genus Bombus (Lee and Dumouchel, 1999) are found in Korea. *B. terrestris* is mass-produced worldwide for use as a pollinator, although *B. ignitus*, dominant in Korea, is substituted for green house pollination (Yoon *et al*., 2003).

Honeybee larvae have been used for the treatment of male impotence and infertility (Meda *et al*., 2004). Bumblebees may also be useful, in the development of nutraceuticals. However, safety evaluation data on these products are scarce and the acute toxicity evaluation of the alcohol extract of *B. ignitus* larvae remains unclear, so that we assessed the acute toxicity of an alcohol extract of *B. ignitus* larvae.

## MATERIALS AND METHODS

***Materials.*** The dried larvae (500 g) of *B. ignitus* was soaked and extracted three times with EtOH by ultrasonification for 30 min. The extracts obtained were dried on a rotary evaporation and freeze-dried as an alcohol extract of *B. ignitus* larvae (BILE).

***Test preparation of BILE.*** Dried alcohol extract of *B. ignitus* larvae were homogenized in a blender to a powder, stored at 4°C, dissolved in phosphate Buffered saline (Sigma-Aldrich Inc., St. Louis, MO), and then orally administered at doses of 0, 0.04, 0.2, 1 or 2 g/kg on a single dose.

***Analysis of mineral content and amino acid composition.*** Mineral content was analyzed by atomic absorption spectroscopy and phosphorus content was determined by colorimetric method, which utilizes ammonium molybdate, hydroquinone, and sodium sulfate (Kim *et al*., 2001). Amino acid Compositional analysis was carried out by derivatization of the first N-terminal amino acids with phenylisothiocyanate (PITC) followed by RP-HPLC (Williams *et al*., 1998).

**Table 1.** Chemical composition and amino acid content of *B. ignitus* larvae extract used in this study (%)

| Component (%) | | | | | | | | Metal content (ppm) | | | |
|---|---|---|---|---|---|---|---|---|---|---|---|
| Protein | Fat | Ash | Ca | P | K | Na | Mg | Fe | Mn | Zn | Cu |
| 40.35 | 23.74 | 4.99 | 0.08 | 0.96 | 1.74 | 0.09 | 0.07 | 89.02 | 9.74 | 58.97 | 12.10 |

| Amino acid | Cys | Met | Asp | Thr | Ser | Glu | Gly | Ala | Pro |
|---|---|---|---|---|---|---|---|---|---|
| Content (%) | 0.250 | 0.670 | 2.583 | 1.310 | 1.424 | 6.032 | 1.970 | 2.169 | 2.805 |
| Amino acid | Val | Ile | Leu | Tyr | Phe | Lys | His | Arg | |
| Content (%) | 1.871 | 1.474 | 2.613 | 1.663 | 1.165 | 1.704 | 0.850 | 1.326 | |

***Animals.*** Specific pathogen free SD rats (5 weeks old, weighing 165 ± 5 g, male and female), purchased from Samtako Co. Ltd. (Osan, Korea), were housed in an environmentally-controlled room with 23 ± 1°C, relative humidity of 55 ± 10%, air ventilation of 10~18 times/hr, a 12-hr light/dark cycle of 150~300 lux, and feed and water available *ad libitum*.

All procedures were conducted in accordance with the Korean Food and Drug Administration (KFDA) "Testing Guidelines for Safety Evaluation of Drugs" (Notification No. 2005-60, issued by the KFDA on Oct 21, 2005). Rats were kept for one week under normal physical conditions (23 ± 2°C, 55 ± 10%, humidity and a regular day/night cycle, air ventilation of 10~18 times/hr, a 12 hr light/dark cycle of 150~300 lux) and fed with standard diet (Samtako Co. Ltd., Osan, Korea), and water *ad libitum* before a single-dose toxicity study testing began.

Five animals of both sexes in each group were weighed, and administered with BILE at a dose of 0.04, 0.2 or 1 or 2 g/kg or its vehicle by a single oral dose treatment.

The test parameters were clinical signs and mortality, body weight, food consumption, hematological analysis, serum biochemical analysis and ophthalmic observation findings (Kim *et al.*, 2002; Ahn *et al.*, 2003).

***Body weight.*** Animals were observed three times daily for clinical signs. Changes in body weight were recorded per three days and group means were calculated. Rat body weights were measured at the initiation of treatment and then per three days until autopsy at 14 days post-treatment initiation.

***Food consumption.*** Daily food consumption was determined by subtracting leftover feed from provided feed. Food consumption was measured daily for the 1$^{st}$ day and per three days thereafter. Again differences between that food supplied and remaining were regarded as daily consumption (g/rat/day).

***Blood sampling and plasma assay.*** After 14 days of treatment, blood (~3 ml) was collected from posterior vena cava under light $CO_2$ inhalation and used for serum chemistry measurements. The parameters examined included total protein, albumin, total bilirubin, glucose, glutamic pyruvic transaminase (GPT), glutamic oxaloacetic transaminase (GOT), alkaline phosphatase (ALP), lactic dehydrogenase (LDH), total cholesterol, blood urea nitrogen (BUN), creatinine, triglyceride, uric acid, sodium, potassium and chloride. All were evaluated using an autoanalyzer (Hitachi 7060 automatic clinical analyzer, Tokyo).

***Statistical analysis.*** Mean and standard errors of all parameters were determined for each of the 5 animals. Student's *t*-test was used to establish the significances of differences between the control and treatment groups. $p < 0.05$ was considered statistically significant.

## RESULTS

***Mineral content of the ethanol extract of B. ignitus lavae.*** The protein fat, ash and mineral content of the larvae ethanol extract from *B. ignitus* were investigated. The larvae extract from *B. ignitus* contained

**Table 2.** Mortality of Sprague-Dawley rats orally treated with *B. ignitus* bee larvae extract on single dose toxicity test

| Sex | Dosage (g/kg) | Days after treatment | | | | | | Final mortality |
|---|---|---|---|---|---|---|---|---|
| | | 0 | 1 | 4 | 7 | 10 | 14 | |
| Female | 0 | 5/5 | 5/5 | 5/5 | 5/5 | 5/5 | 5/5 | 0/5 |
| | 0.04 | 5/5 | 5/5 | 5/5 | 5/5 | 5/5 | 5/5 | 0/5 |
| | 0.2 | 5/5 | 5/5 | 5/5 | 5/5 | 5/5 | 5/5 | 0/5 |
| | 1 | 5/5 | 5/5 | 5/5 | 5/5 | 5/5 | 5/5 | 0/5 |
| | 5 | 5/5 | 5/5 | 5/5 | 5/5 | 5/5 | 5/5 | 0/5 |
| Male | 0 | 5/5 | 5/5 | 5/5 | 5/5 | 5/5 | 5/5 | 0/5 |
| | 0.04 | 5/5 | 5/5 | 5/5 | 5/5 | 5/5 | 5/5 | 0/5 |
| | 0.2 | 5/5 | 5/5 | 5/5 | 5/5 | 5/5 | 5/5 | 0/5 |
| | 1 | 5/5 | 5/5 | 5/5 | 5/5 | 5/5 | 5/5 | 0/5 |
| | 5 | 5/5 | 5/5 | 5/5 | 5/5 | 5/5 | 5/5 | 0/5 |

more proteins in following proportions: protein (40.4%), Ca (0.08%), P (0.96%), K (1.74%), Na (0.09%), Mg (0.07%), Fe (89.02 ppm), Mn (9.74 ppm), Zn (58.97 ppm) and Cu (12.10 ppm) (Table 1). Amino acid content of larvae extract from *B. ignitus* showed no obvious trend in the changes of protein and mineral contents in the statistical perspective (Table 1).

***Clinical signs and food consumption.*** No deaths or adverse clinical signs were observed due to the ingestion of BILE at doses of 0.04, 0.2, 1.0 or 2.0 g/kg (Table 2). Food consumption levels were similar for all study groups, though it was more or less differences between control and treated groups (Fig. 1).

***Body weight changes.*** There were no toxicologically significant differences in mean body weight between any of the treatment groups (Fig. 2). During the 14 day administration period after a single dose treatment, the body weights of the male and female SD rats in the 4 treatment groups were comparable across the control and treated groups. The mean per 2 days body weights versus time are presented in Fig. 2. In the male rats, there were statistically significant differences in body weight between the BILE treated groups (0.04 g/kg, 1.0 g/kg, or 2.0 g/kg body weight gain increase compared control) and the control group ($p > 0.05$); however, no statistically significant differences were observed between the 0.2 g/kg BILE treated group and the control group. But, no significant differences in body weight gain were observed in the female BILE treated rats.

***Hematology and blood chemistry.*** Minor changes in male rats were found in hematological parameters for the 0.04 g/kg, 0.2 g/kg, 1 g/kg and 2 g/kg larvae extract from *B. ignitus* treated groups (total cholesterol increase of 11.5~34.5%, potassium serum level reduction of 30.8~64.7% and increase of sodium level of 4.6~7.1%), but all changes were within physiological range (Table 3 and Table 4). In the sera of the BILE treated

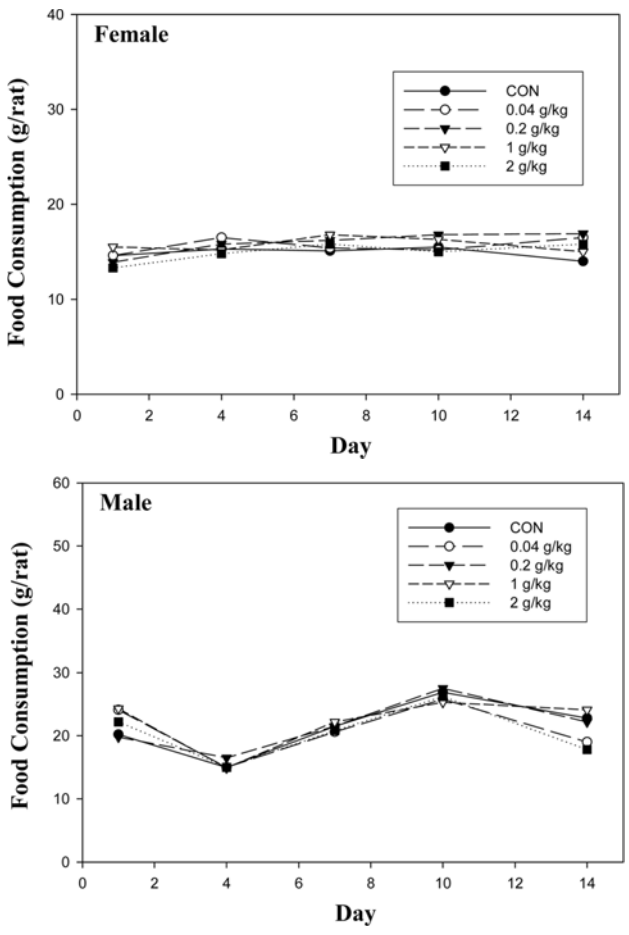

**Fig. 1.** Food consumption of male and female SD rats, treated orally with *B. iginitus* larvae extract on a single dose.

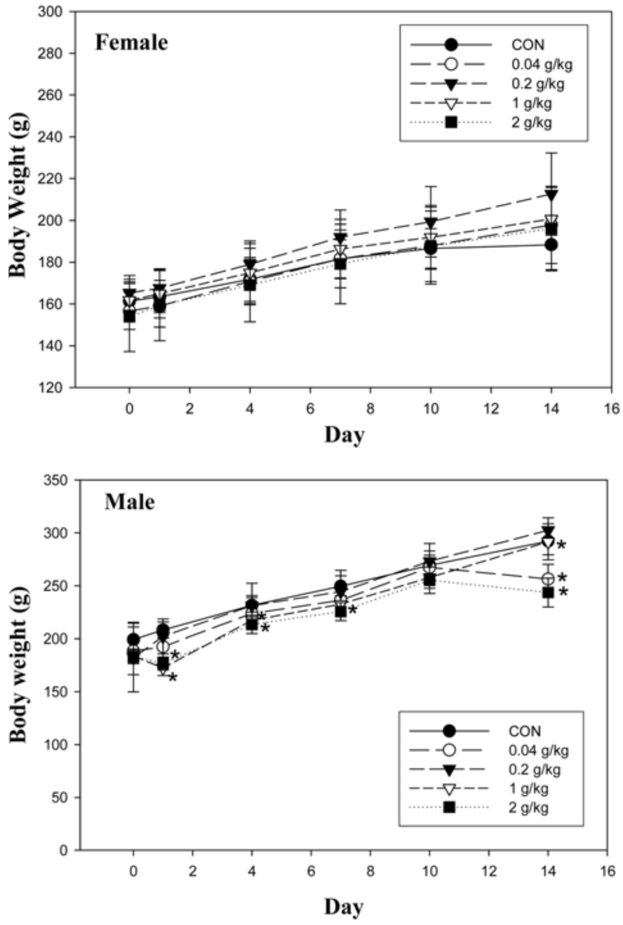

**Fig. 2.** Body weight increases of male and female SD rats, treated orally with *B. iginitus* larvae extract on a single dose. *Significantly different from the untreated controls ($p < 0.05$).

**Table 3.** Biochemical serum values of female rats treated orally with *B. ignitus* bee larvae extract

| Item | g/kg | CON[a] | 0.04 | 0.2 | 1 | 2 |
|---|---|---|---|---|---|---|
| Toal protein | g/dl | 6.9 ± 0.6 | 6.4 ± 0.4 | 6.5 ± 0.4 | 6.1 ± 0.4 | 6.9 ± 0.4 |
| GPT | IU/l | 78.0 ± 44.3 | 73.3 ± 42.4 | 45.5 ± 3.5 | 64.6 ± 47.4 | 57.3 ± 13.6 |
| GOT | IU/l | 176.5 ± 23.3 | 170.3 ± 59.2 | 110.5 ± 2.1 | 214.0 ± 17.6 | 180.3 ± 19.3 |
| ALP | IU/l | 253.3 ± 107.2 | 199.3 ± 53.0 | 209.5 ± 119.5 | 207.6 ± 75.9 | 346.7 ± 62.5 |
| Glucose | mg/dl | 202.3 ± 125.9 | 203.3 ± 86.0 | 167.0 ± 25.5 | 199.8 ± 53.4 | 266.5 ± 116.5 |
| T-Cholesterol | mg/dl | 92.7 ± 18.4 | 77.3 ± 13.6 | 79.5 ± 0.7 | 74.2 ± 10.6 | 85.6 ± 3.5 |
| Bilirubin | mg/dl | 0.3 ± 0.1 | 0.2 ± 0.1 | 0.1 ± 0.0 | 0.1 ± 0.1 | 0.2 ± 0.1 |
| BUN | mg/dl | 24.1 ± 7.0 | 23.0 ± 3.4 | 23.3 ± 2.3 | 19.7 ± 2.6 | 21.8 ± 2.2 |
| Creatine | mg/dl | 0.6 ± 0.1 | 0.6 ± 0.2 | 0.5 ± 0.0 | 0.5 ± 0.1 | 0.7 ± 0.1 |
| Triglyceride | mg/dl | 154.3 ± 70.3 | 104.7 ± 46.5 | 83.0 ± 25.5 | 74.4 ± 18.3 | 200.0 ± 73.9 |
| Uric acid | mg/dl | 5.5 ± 3.4 | 5.5 ± 0.7 | 5.4 ± 0.7 | 4.5 ± 1.4 | 10.4 ± 5.6 |
| K | mmol/l | 19.1 ± 4.8 | 21.6 ± 1.4 | 18.8 ± 4.7 | 17.2 ± 4.1 | 21.8 ± 0.9 |
| Na | mmol/l | 139.7 ± 6.1 | 137.3 ± 1.5 | 139.0 ± 5.7 | 139.0 ± 4.6 | 136.0 ± 3.0 |
| Ca | mmol/l | 11.2 ± 1.1 | 10.6 ± 0.9 | 10.8 ± 0.4 | 9.9 ± 0.5* | 12.8 ± 1.5 |
| CK | IU/l | 696.0 ± 15.0 | 761.7 ± 114.7 | 490.5 ± 34.6 | 575.8 ± 292.8 | 838.7 ± 272.8 |
| LDH | IU/l | 3855.3 ± 2375.5 | 3223.7 ± 675.1 | 1917.5 ± 3.5 | 3248.4 ± 1791.9 | 2243.7 ± 816.6 |
| HDL | IU/l | 26.0 ± 2.7 | 21.7 ± 3.1 | 24.0 ± 1.4 | 22.4 ± 3.7 | 24.3 ± 2.1 |

[a]CON: PBS (vehicle) treated with murine normal diet.
Each value represents mean ± S.D.
Statistically significant from control (**p* < 0.05, ***p* < 0.01).

**Table 4.** Biochemical serum values of male rats treated orally with *B. ignitus* bee larvae extract

| Item | g/kg | CON[a] | 0.04 | 0.2 | 1 | 2 |
|---|---|---|---|---|---|---|
| Toal protein | g/dl | 6.5 ± 0.1 | 6.9 ± 0.6 | 6.7 ± 0.4 | 6.3 ± 0.2 | 6.7 ± 0.3 |
| GPT | IU/l | 61.0 ± 4.2 | 53.3 ± 5.1 | 59.2 ± 7.8 | 64.3 ± 4.6 | 49.8 ± 4.4* |
| GOT | IU/l | 136.5 ± 20.5 | 112.0 ± 14.7 | 147.5 ± 23.8 | 138.3 ± 40.2 | 94.4 ± 14.8 |
| ALP | IU/l | 352.0 ± 58.0 | 440.7 ± 40.5 | 484.5 ± 88.5 | 384.0 ± 39.1 | 406.4 ± 68.7 |
| Glucose | mg/dl | 325.5 ± 95.5 | 309.7 ± 174.7 | 303.8 ± 74.1 | 262.0 ± 100.0 | 351.4 ± 81.6 |
| T-Cholesterol | mg/dl | 65.0 ± 1.4 | 87.7 ± 2.1** | 74.8 ± 6.5 | 78.3 ± 2.1* | 80.8 ± 9.3* |
| Bilirubin | mg/dl | 0.1 ± 0.0 | 0.1 ± 0.0 | 0.1 ± 0.0 | 0.1 ± 0.0 | 0.1 ± 0.0 |
| BUN | mg/dl | 19.7 ± 2.0 | 27.3 ± 3.9 | 24.2 ± 5.1 | 22.9 ± 2.5 | 25.4 ± 2.0* |
| Creatine | mg/dl | 0.7 ± 0.1 | 0.6 ± 0.1 | 0.7 ± 0.1 | 0.7 ± 0.1 | 0.6 ± 0.1 |
| Triglyceride | mg/dl | 181.0 ± 58.0 | 148.3 ± 45.2 | 147.8 ± 47.2 | 109.0 ± 17.9. | 155.2 ± 59.1 |
| Uric acid | mg/dl | 8.9 ± 3.3 | 7.0 ± 2.7 | 7.5 ± 1.3 | 5.7 ± 2.1 | 8.8 ± 3.2 |
| K | mmol/l | 22.1 ± 0.2 | 15.3 ± 0.8* | 14.4 ± 0.8** | 8.9 ± 2.4* | 7.8 ± 1.5* |
| Na | mmol/l | 140.0 ± 1.4 | 145.0 ± 2.6 | 146.4 ± 0.9** | 149.5 ± 2.1* | 150.0 ± 2.1* |
| Ca | mmol/l | 11.9 ± 0.7 | 11.9 ± 1.5 | 12.2 ± 0.7 | 11.5 ± 1.1 | 12.8 ± 0.9 |
| CK | IU/l | 775.5 ± 491.4 | 434.0 ± 296.8 | 588.8 ± 172.1 | 392.7 ± 153.9 | 334.4 ± 219.7 |
| LDH | IU/l | 2158.0 ± 475.2 | 1514.3 ± 1060.1 | 2171.0 ± 323.2 | 1735.3 ± 886.3 | 1116.6 ± 934.1 |
| HDL | IU/l | 22.0 ± 1.4 | 27.0 ± 1,0* | 23.3 ± 2.2 | 21.0 ± 1.8 | 26.6 ± 2.9 |

[a]CON: PBS treated with murine normal diet.
Each value represents mean ± S.D.
Statistically significant from control (**p* < 0.05, ***p* < 0.01).

groups, the level of potassium ion in male was significantly lower than in the control. A significant increase in the total cholesterol was observed in the 0.04 g/kg, 1.0 g/kg and 2.0 g/kg group for only the males (controls, 65.0 ± 1.4 mg/dl; 0.04 g/kg. 87.7 ± 2.1 mg/dl; 1.0 g/kg, 78.3 ± 2.1 mg/dl; 2.0 g/kg, 80.8 ± 9.3 mg/dl). The potassium ion levels of the treated groups were reduced in dose dependent manners in the males (control, 22.1 ± 0.2 nmol/l; 0.04 g/kg, 15.3 ± 0.8 nmol/l; 0.2 g/kg, 14.4 ± 0.8 nmol/l, 1.0 g/kg: 8.9 ± 2.4 nmol/l; 2.0 g/kg: 7.8 ± 1.5 nmol/l), but not reduced significantly in females (controls, 19.1 ± 4.8 nmol/l; 2.0 g/kg, 21.8 ± 0.9 nmol/l). Whereas the sodium ion level increased (male: control, 140.0 ± 1.4 nmol/l; 0.2 g/kg, 146.4 ± 0.9 nmol/l; 1.0 g/kg, 149.5 ± 2.1 nmol/l; 2.0 g/kg, 150.0 ± 2.1 nmol/l), but did not increase in female treated groups (Table 3 and Table 4).

Some significant differences were observed between the treated and control groups with respect to the hematological parameters at the end of the experiment. At the end of the administration period, as a coagulation parameter, no increase in platelet count was observed in the male and female rats in all of the treated groups; as an indicator of RBC function, we found that MCV, MCH and MCHC were not significantly different between the treated groups and the control group (data not shown).

***Ophthalmologic findings.*** No significant treatment-related ophthalmologic findings were observed. Any minor changes were few and dose independent.

## DISCUSSION

In ancient times, people in Oriental countries, especially Japan and Taiwan, used bee larvae as a crude drug or protein source during food shortages: now bumblebees are mass-produced year-round. To investigate the safety of using bumblebee products as domestic foods or medicinal drugs with the potential for prevention and curing efficacy, we evaluated the toxicity profile of single dose oral treatment of SD rats with the ethanol extract of Bumblebee larvaes. *B. ignitus* larvae alcohol extract (BILE) did not induce any remarkable acute toxic responses and there were no significant changes in diet consumption, ophthalmologic findings, clinical pathology (hematology, clinical chemistry, coagulation, and urinalysis), or gross pathology up to 2 g/kg. The potassium levels in $14^{th}$ day-rat serum after single-dose treatment was reduced in a dose dependent manner in male rats; the potassium level of the extract was higher (1.74%) than other cation level (Ca, 0.08%; Na, 0.09%; Ma, 0.07%). These results, however, were not sufficient to deem BILE a diuretic agent (Furukawa *et al.*, 1997) but the data obtained can be interpreted within the context of a *B. ignitus* extract preparation study as a medicine or food (Hu *et al.*, 2009). In male rats, there were statistically significant differences in body weight gain between the BILE (0.04 g/kg; 1.0 g/kg; 2.0 g/kg) treated groups and the control group (group, $p > 0.05$); however, no statistically significant differences were observed between BILE-treated group and the control group in the female rats. Therefore, the approximate lethal oral dose of the ethanol extract from larvae of *B. ignitus* was concluded to be higher than 2 g/kg in rats.
